# Does Fatigue Complaint Reflect Memory Impairment in Multiple Sclerosis?

**DOI:** 10.1155/2014/692468

**Published:** 2014-03-02

**Authors:** Caroline Jougleux-Vie, Emeline Duhin, Valerie Deken, Olivier Outteryck, Patrick Vermersch, Hélène Zéphir

**Affiliations:** ^1^Department of Neurology, Roger Salengro Hospital, 59037 Lille, France; ^2^Department of Biostatistics and INSERM EA 2694, Lille University Hospital and University of Lille North of France, 59037 Lille, France

## Abstract

*Background and Purpose*. Fatigue and memory impairment are common symptoms in multiple sclerosis (MS) and both may interact with cognition. This can contribute to making a complaint misrepresentative of the objective disorder. We sought to determine whether fatigue complaint in MS reflects memory impairment and investigated whether patients' subjective fatigue is associated with memory complaint. *Methods*. Fifty MS patients complaining of fatigue underwent subjective assessment of fatigue and memory complaint measured using self-assessment scales. Cognitive functions were assessed using a battery of neuropsychological tests, including a test of verbal episodic memory, the selective reminding test (SRT). Correlations were studied between subjective fatigue, memory complaint, and performance in verbal episodic memory. *Results*. Depression score, psychotropic and/or antiepileptic drug use, Expanded Disability Status Scale (EDSS) score, and MS form were confounding factors. After adjusting for these confounding factors, neither fatigue complaint nor memory complaint was correlated with SRT performance. Subjective fatigue was significantly associated with memory complaint. *Conclusion*. Although complaint of fatigue in MS was correlated with memory complaint, subjective fatigue was not the expression of memory impairment.

## 1. Introduction

Multiple sclerosis (MS) is a chronic inflammatory demyelinating disease of the central nervous system, characterized by various levels of physical, emotional, cognitive, and social difficulties. Fatigue, cognitive impairment, and depression are common in MS [1]. Fatigue is reported to occur in 53% to 83% of MS patients [2]. Thirty to fifty-five percent of MS patients consider fatigue as the most disabling symptom [3]. Fatigue can occur in all MS forms at any stage and is reported to impact employment in 41% and social functioning in 25% of MS patients [3]. Three dimensions of fatigue are described: cognitive, physical, and psychosocial, related to the impact of fatigue on cognitive, physical, and psychosocial functioning, respectively [4, 5].

The prevalence of depression in MS is reported to range from 16% to 46% and depression is considered to be more frequent in MS than in other chronic neurological diseases, with a higher risk of suicide [6–8]. Cognitive impairment is present in 40% to 65% of MS patients at any stage of the disease [9, 10] and mainly affects long-term episodic and working memory, executive functions, information processing speed, and attention. Verbal episodic memory impairment and memory complaint are common in the early stage of the disease. Among previous studies that examined the relationship between fatigue, cognition, and mood disorders, some found a link between depression and cognitive impairment [11] whereas others did not [12, 13]. Although a relationship has been demonstrated between fatigue and depression [14], the relationship between fatigue and cognitive impairment is still debated. Indeed, cognitive fatigue, defined as a decline of cognitive performance during a sustained cognitive task, seems to be correlated with cognitive impairment, whereas subjective fatigue is not associated with cognitive impairment [15].

Cognitive complaint seems to be close to psychic factors, such as depression, fatigue, and anxiety, and is not correlated with an objective cognitive deficit evaluated by neuropsychological assessment [16].

Such interactions between cognitive dysfunction, dysthymia, and fatigue make it unlikely that patients are able to perceive the real difficulty. Moreover, the patient might be unable to distinguish and identify the right disorder. The primary objective of our study was to determine whether fatigue complaint in MS could reflect memory impairment. The secondary objective was to investigate whether patients' subjective fatigue is associated with memory complaint.

## 2. Materials and Methods

### 2.1. Patients

We consecutively included 50 MS patients who complained of fatigue between August 2011 and May 2012. MS was defined according McDonalds' 2005 criteria [17]. Fatigue was defined as a chronic fatigue that has occurred during at least six months and for more than half of that time [18]. Exclusion criteria were (i) a history of any clinically relevant psychiatric disease or other neurological diseases, (ii) a history of drug or alcohol abuse, (iii) a relapse, steroid treatment, or recent onset of a new disease modifying drug during the month preceding the study, and (iv) difficulty in understanding French. Details of MS patients' demographic and clinical characteristics and treatments were retrospectively extracted from medical files and according to a standardized questionnaire. Patients gave their written, informed consent to the study. The study was approved by the local ethics committee.

### 2.2. Outcome Criteria

Subjective fatigue was assessed using the Fatigue Impact Scale [19] (FIS, French validated version), a 40-item autoevaluation scale, each item being scored from 0 (no problem) to 4 (extreme problem), providing a continuous scale from 0 to 160. It is made up of four subscales that describe how fatigue impacts on cognitive (10 items), physical (10 items), psychosocial (10 items), and social functioning (10 items). The score is a reflection of functional limitations due to fatigue experienced within the previous month rather than a measure of the level of fatigue. A higher score indicates more reported fatigue.

Subjective memory complaint was evaluated by the 64 items of the Questionnaire Autoévaluation de la Mémoire [20] (QAM), classified into 10 categories to assess memory lapses concerning conversations, films and books, people, and general knowledge. Each item is scored from 1 to 6 (1: never, 2: rarely, 3: sometimes, 4: often, 5: very often, and 6: always). A mean score is calculated for each category.

To evaluate depressive symptoms, patients completed the French validated version of the 21-item Beck Depression Inventory (BDI-II) [21]. Each item is scored from 0 to 3 and the sum of all items gives the total BDI-II score ranging from 0 to 63. Scores between 0 and 13 define a minimal depressive state, between 14 and 19 a slight depressive state, between 20 and 28 a moderate depressive state, and between 29 and 63 a high depressive state.

Every scale was performed by the patients themselves 2 weeks before the neuropsychological testing in order not to interfere with cognitive fatigue.

### 2.3. Neuropsychological Assessment

Neuropsychological testing focused on the cognitive domains that are commonly impaired in MS. The neuropsychological tests battery was administered by a neuropsychologist in 1 hour. The battery, named BCcogSEP [22], is a modified version of “the Brief Repeatable Battery of Neuropsychological tests for Multiple Sclerosis” (BRB-N) and was administered in a French version. The BCcogSEP is made up of 8 tests as listed hereafter. (1) The selective reminding test (SRT) measures verbal episodic memory, with 3 scores considered: mean number of words in immediate recall, learning index, and delayed recall [23]. (2) The 10/36 spatial recall test assesses visual learning (10/36) [10]. (3) The symbol digit modality test (SDMT) measures information processing speed and selective attention [24]. (4) The crossed tapping test evaluates mental flexibility [25]. (5) The paced auditory serial addition test (PASAT) evaluates sustained attention, information processing speed, and working memory [26]. (6) Memory span assesses immediate and working memory [22]. (7) The word list generation test measures semantic and phonemic verbal fluency [22]. (8) The GOnoGO test assesses response inhibition [27].

Fourteen scores were obtained and a patient was considered impaired when a score was inferior to percentile 5 compared to the normal range. A patient was considered to present global cognitive damage when at least 4 scores were lower than percentile 5 [22].

### 2.4. Statistical Analysis

Statistical analyses were performed using SAS software, version 9.2 (SAS Institute Inc., Cary, NC, USA). The level of significance was set at 5%. Numerical variables are expressed as mean ± standard deviation (SD) and qualitative parameters as frequency and percentage.

For the primary objective, the outcome measurements were to assess the relationship between the 3 scores of the SRT (number of words, SRT learning index, and delayed recall), the score of information processing speed and the global cognitive performance and each of the following 4 independent variables separately: total FIS score, FIS cognitive subscore, FIS physical subscore, and the QAM (independent variables). First, the influence of the clinical and therapeutic characteristics on the dependent variables (the 3 scores of the SRT and QAM) was studied by bivariate analyses. The Mann-Whitney *U* test was used for qualitative binary values (gender, occupational status, presence of autoimmune comorbidities, form of MS, and association with psychotropic and/or antiepileptic drugs) and the Kruskal-Wallis test for evaluating qualitative values in more than 2 classes (the treatment for MS). The correlation with quantitative variables (disease duration, Expanded Disability Status Scale (EDSS), age, and BDI-II) was assessed by a Pearson correlation coefficient test. For each dependent variable, parameters with a *P* value less than 0.1 were considered as confounding factors for subsequent analyses.

In a second stage, the correlation between each dependent variable and each of the 4 independent variables separately (total FIS score, FIS cognitive subscore, FIS physical subscore, and the QAM) was computed with a Pearson correlation coefficient test. For each dependent variable (e.g., SRT number of words) and each independent variable (e.g., total FIS score), a linear regression was performed in order to adjust for the confounding factors.

The same analysis was performed with QAM as dependent variable and the 3 FIS scores separately (total FIS score, the FIS cognitive subscore, and the FIS physical subscore) as independent variables.

## 3. Results

### 3.1. Demographic and Clinical Characteristics of Included Patients (Table 1)

Fifty MS patients were included in the analysis. The sex ratio showed a predominance of women (66%) in the patient group. The mean education level was 12 ± 2 years; the median EDSS score was 3.0 [2.0–5.0], and the mean disease duration was 13 ± 7.9 years. The mean depression score was 18.76 ± 11.55. The mean fatigue score was 95.4 ± 30. The mean scores for each fatigue dimension, that is, cognitive, physical, psychosocial, and social functioning scores, are reported in Table 2. The physical dimension of the fatigue was the most frequent complaint.

Concerning memory complaint, the mean QAM score was 2.75 ± 0.84. A memory complaint in at least one dimension of the QAM was observed in 58% of MS patients (*n* = 29). Twenty-two (44%) MS patients presented cognitive impairment. Working memory impairment was the most frequent (62%, *n* = 31), followed by executive deficits (60%, *n* = 30). Attentional and information processing speed deficits were observed in 29 patients (58%). Sixteen patients (32%) presented a verbal episodic memory deficit on the three scores of the SRT: number of words reminded, learning index, and delayed recall (Table 3).

### 3.2. Correlations between Fatigue, Memory Complaint and Verbal Episodic Memory, Information Processing Speed, and Global Cognitive Performances

Depression, EDSS status, concomitant treatment (psychotropic and/or antiepileptic treatment), MS form (progressive form), and MS treatments interfered with subjective fatigue and memory complaint scores and verbal episodic memory performance (Table 4). We verified that after adjustment for mood there was no link between any of the concomitant treatment classes (such as benzodiazepines, antiepileptics, or antidepressants) and fatigue and memory performances. Moreover, after adjustment for mood, the EDSS score was significantly associated with the physical subscore of fatigue (*P* = 0.048). After adjustment for confounding factors (Table 4), verbal episodic memory performance, assessed by the number of words recalled, learning, and delayed recall, was not correlated with either the total score (number of words *P* = 0.1204, learning *P* = 0.3216, and delayed recall *P* = 0.6389), the cognitive subscore (number of words *P* = 0.1444, learning *P* = 0.1249, and delayed recall *P* = 0.5790), and the physical subscore (number of words *P* = 0.1511, learning *P* = 0.4114, and delayed recall *P* = 0.7430) of the FIS or memory complaint (number of words *P* = 0.4297, learning *P* = 0.2569, and delayed recall *P* = 0.3828). Information processing speed was not correlated with either the total score (*P* = 0.31) or the cognitive and physical subscore (*P* = 0.65 and *P* = 0.14, resp.). Global cognitive performance was not correlated with either the total score (*P* = 0.93) or the cognitive and physical subscore (*P* = 0.21 and *P* = 0.86, resp.). Both the total score (*P* < 0.0001) and the cognitive subscore (*P* < 0.0001) of the subjective fatigue scale were correlated with memory complaint. There was no significant link between physical subscore of subjective fatigue and memory complaint (*P* = 0.053).

## 4. Discussion

Our findings show that subjective fatigue complaint was associated with memory complaint but not with memory performances.

Our study did not find any correlation between subjective fatigue in MS patients and verbal episodic memory performance evaluated by the SRT test and global cognitive performance evaluated by the number of scores impaired on the BCcogSEP. Moreover, subjective fatigue was not correlated with information processing speed, a cognitive function frequently impaired in MS patients. In the literature, conflicting results have been reported concerning a correlation between fatigue complaint and episodic verbal memory performance and between fatigue complaint and information processing speed. Nocentini et al. reported that fatigue complaint on the Fatigue Severity Scale (FSS) was correlated with immediate and delayed recall scores on the 15-word Rey's test [28]. Mattioli et al. showed a correlation between the FIS score and the delayed recall score on the SRT [29]. Diamond et al. showed that slower information processing was correlated with higher levels of fatigue [12]. However, in contrast to our study, these results were not adjusted for confounding factors. According to the literature, the intensity of fatigue complaint in MS patients does not reflect their memory performance. In previous studies, the degree of fatigue, as defined by the FSS or FIS scales, was not significantly associated with Paired Associate Learning Test (PALT) scores, evaluating episodic verbal memory [30]. In the study by Krupp and Elkins, a decrease in memory performance on the SRT test was not correlated with an increase of subjective fatigue [31]. More generally, no correlation was found between cognitive performance and subjective fatigue [15]. Moreover, our results did not show a correlation between memory complaint and episodic verbal memory performance. MS patients might have a poor ability for self-assessment of their memory difficulty. However, this memory complaint in our results reflects subjective fatigue complaint and it therefore seems necessary to assess fatigue when memory complaint occurs. In our study, a high percentage of the MS patients had a high score on the FIS and the physical dimension of the fatigue was the most frequent complaint. This physical dimension is more pronounced in MS patients with a higher EDSS score because EDSS assesses mainly the physical disability of MS. As in a study by Pittion-Vouyovitch et al., the physical dimension was more involved than the other dimensions of fatigue [32]. Forty-four percent of MS patients in our study presented a cognitive impairment, a similar proportion to that found in other studies [10, 28]. There is a variability in the occurrence of memory performance disturbances reported in the literature, ranging from 29% to 69% of MS patients. Such variability is likely due to the different tools used to test verbal episodic memory [33]. In our study, 32% of MS patients presented verbal episodic memory impairment and, as expected, MS patients with a progressive form presented higher memory impairment than relapsing MS patients [34].

## 5. Conclusion 

Our study found that fatigue was not related to impairment of objective memory performance but could affect MS patients' perception regarding their memory. Memory complaint of MS patients was associated not with their memory performance but with their fatigue.

## Figures and Tables

**Table 1:** Clinical and demographic characteristics of MS patients.

	MS patients (*n* = 50)
Females *n* (%)	37 (74)
Males, *n* (%)	13 (26 )
Education level, mean years ± SD	12 ± 2
Professional activity, *n* (%)	23 (46)
MS form, *n* (%)	
RR	35 (70)
PP	10 (20)
SP	5 (10)
Disease duration, mean years, ± SD	13 ± 8
EDSS score, median (IQR) (25–75)	3.0 (2.0–5.0)
MS treatments, *n* (%)	
No treatment	16 (32)
Injectable immunomodulators	15 (30)
Natalizumab	12 (24)
Other immunosuppressants	7 (14)
Concomitant treatments, *n* (%)	
Benzodiazepine	11 (22)
Antidepressant	12 (24)
Hypnotic	1 (2)
Antiepileptic	11 (22)
BDI-II score, median (IQR) (25–75)	14.5 (10–26)
Depressive state, *n* (%)	
Minimal	9 (18)
Slight	20 (40)
Moderate	10 (20)
High	11 (22)

BDI-II: Beck Depression Inventory; EDSS: Expanded Disability Status Scale; IQR: interquartile range; *n*: number of patients; MS: multiple sclerosis; RR: relapsing remitting; PP: primary progressive; SD: standard deviation; SP: secondary progressive.

**Table 2:** Fatigue scale and memory complaint scale scores.

	Mean score ± SD
FIS (French validated version)	
Total score (/160)	95.4 ± 30
Cognitive subscore (/40)	22.2 ± 9.8
Physical subscore (/52)	36.4 ± 11.2
Psychosocial subscore (/52)	27 ± 10.8
Social functioning subscore (/16)	9.8 ± 4
QAM score	2.75 ± 0.84

FIS: Fatigue Impact Scale; QAM: Questionnaire d'Autoévaluation de la Mémoire; SD: standard deviation.

**Table 3:** Proportion of MS patients with impaired cognitive dysfunction, assessed using BCcogSEP.

	*n* (%)
Cognitive impairment (≥4/14 scores impaired)	22 (44)
SRT	16 (32)
Number of words recalled	11 (22)
Learning index	11 (22)
Delayed recall	11 (22)
10/36	9 (18)
Total recall	3 (6)
Delayed recall	8 (16)
Auditory verbal spans	23 (46)
Direct span	17 (34)
Indirect span	17 (34)
SDMT	20 (40)
PASAT	23 (46)
3 sec	20 (40)
2 sec	16 (32)
Crossed tapping	10 (20)
GOno/GO	6 (12)
Verbal fluency	25 (50)
Phonemic	22 (44)
Semantic	9 (18)

BCcogSEP: Batterie Courte d'Evaluation des fonctions Cognitives des patients ayant une Sclérose en Plaques; *n*: number of patients; PASAT: paced auditory serial addition test; SDMT: symbol digit modality test; SRT: selective reminding test.

**Table 4:** Correlations between the clinical and therapeutic characteristics and the SRT, QAM, and FIS scores.

	QAM	FIS			SRT		
		Total score	Cognitive subscore	Physical subscore	Number of words recalled	Learning index	Delayed recall
MS form	0.80	0.46	0.49	0.004*	0.12	0.01*	0.03*
EDSS score	0.15	0.007*	0.18	0.0002*	0.05	0.002*	0.02*
Concomitant treatment	0.02*	0.03*	0.13	0.008*	0.01*	0.01*	0.01*
BDI-II	0.0002*	<0.0001*	<0.0001*	0.0006*	0.07	0.08	0.01*
Age	0.63	0.55	0.81	0.40	0.54	0.09	0.73
Sex	0.43	0.55	0.31	0.95	0.1	0.62	0.35
Education level	0.56	0.68	0.67	0.81	0.18	0.06	0.11
Professional activity	0.74	0.54	0.84	0.40	0.38	0.07	0.09
Other autoimmune disorders	0.16	0.11	0.06	0.33	0.82	0.33	0.38
MS treatments	0.10	0.26	0.96	0.01*	0.84	0.28	0.46
Disease duration	0.91	0.85	0.82	0.82	0.83	0.72	0.55

*P values < 0.05 are shown in bold; BDI-II: Beck Depression Inventory; EDSS: Expanded Disability Status Scale; FIS: Fatigue Impact Scale; MS: multiple sclerosis; QAM: Questionnaire d'Autoévaluation de la Mémoire; SRT: Selective Reminding Test.
